# Reduction in metabolic noise reveals rejuvenation following transient severe caloric restriction

**DOI:** 10.1007/s11357-023-00969-1

**Published:** 2023-11-10

**Authors:** Guy Levkovich, Inna Bendikov-Bar, Sergey Malitsky, Maxim Itkin, Mark Rusal, Dmitri Lokshtanov, Dmitry Shinder, Dror Sagi

**Affiliations:** 1 Institute of Animal Science, Department of Poultry and Aquaculture, Agricultural Research Organization, Volcani Center, Rishon LeZion, Israel; 2 The Mina and Everard Goodman Faculty of Life Sciences, The Sagol Center for Healthy Human Longevity, Bar-Ilan University, Ramat Gan, Israel; 3 Life Sciences Core Facilities, Weizmann Institute of Science, Rehovot, Israel

**Keywords:** Laying hens, Systems biology, Metabolomics, Molting, Aging

## Abstract

Among land vertebrates, the laying hen stands out due to its great reproductive efficiency: producing an egg daily all year long. This production rate makes the laying hen a special model animal to study the general process of reproduction and aging. One unique aspect of hens is their ability to undergo reproductive plasticity and to rejuvenate their reproductive tract during molting, a standard industrial feed restriction protocol for transiently pausing reproduction, followed by improved laying efficiency almost to peak production. Here we use longitudinal metabolomics, immunology, and physiological assays to show that molting promotes reproduction, compresses morbidity, and restores youthfulness when applied to old hens. We identified circulating metabolic biomarkers that quantitatively predict the reproduction and age of individuals. Lastly, we introduce metabolic noise, a robust, unitless, and quantifiable measure for heterogeneity of the complete metabolome as a general marker that can indicate the rate of aging of a population. Indeed, metabolic noise increased with age in control hens, whereas molted hens exhibited reduced noise following molting, indicating systemic rejuvenation. Our results suggest that metabolic noise can be used as a quick and universal proxy for assessing successful aging treatments, accelerating the timeline for drug development.

**Supplementary Information:**

The online version contains supplementary material available at 10.1007/s11357-023-00969-1.

## Introduction

The laying hen provides a special model for aging and reproduction, as it produces an egg daily all year long [1, 2]. Such laying efficiency provides a unique and reliable assay to observe reproductive differences even between individuals of the same age and genetic background.

One unique aspect of hens is their ability to rejuvenate their reproductive tract during molting [3], a severe and acute feed restriction protocol [4, 5] developed by farmers to extend the profitable period of the flock. The procedure is characterized by weight loss, shedding of feathers, and complete cessation of egg production. Following the molt, the animals gradually return to *ad libitum* feeding, regrow their feathers, and exhibit remarkable reproductive plasticity: egg production is resumed almost to maximum, significantly higher than before the procedure [4]. The importance of molting to agriculture has led to an abundance of research that focuses on its detailed regulation in the context of animal productivity. However, the explicit effects of this procedure on aging remain unclear.

This study combined physiological assays, metabolic profiling [6, 7], and cytokines arrays as a non-invasive methodology to quantify the systemic effect of molting on senesce and reproductive aging. Computational and machine learning schemes identified metabolic biomarkers that accurately and quantifiably predicted metabolic age and reproduction. Together, the molecular and physiological data indicated that molting systemically slowed down aging and even rejuvenated the hens. Furthermore, the aging biomarkers revealed metabolic changes in the molted cohort that are associated with the general process of aging, rather than the specific process of molting in hens.

We then applied metabolic noise, a quantifiable measure of the heterogeneity of the entire metabolome, as a generic and system-independent biomarker of aging [6]. The reliability and robustness of metabolic noise were utilized using molting as a reference anti-aging treatment.

Indeed, the heterogeneity of control animals increased with age, as it represents an increase in population variability or entropy with time. However, molting resulted in reduced metabolic noise, indicating reduced population variability and rejuvenating the hens. Metabolic noise, therefore, could be used as a quick and universal proxy for successful aging treatments, shortening the timeline for anti-aging drug development.

## Methods

### Animal husbandry

White Leghorn (Lohman) laying hens, all females, were purchased from commercial husbandries (Hasolelim, Israel) at the age of 1 day and raised in the poultry farm of the Volcani Center, Israel. Maintenance conditions and feeding formulas were according to the Lohman guidelines (https://hylinena.com/wp-content/uploads/2019/10/Lohmann_LSL-Lite16-2.pdf), with free accesses to food and water.

Throughout the study, hens were accommodated in Individual cages (40 × 40 × 43 cm), one hen per cage, to allow longitudinal tracking of egg production, egg weight, and egg quality.

### Molting protocol

We used a standard molting protocol used in commercial chicken facilities and is typically applied around 18 months of age when the flock becomes commercially unsustainable due to reproductive aging.

Molting protocol included food deprivation for 10 days, until the hens lost about 30% of body weight (down to 1300 g). We then let them recover for 11 more days under 120 g molting mixture every other day. The molting mixture is low in protein and calories, so it does not support egg production, and only allows safe recovery from the fasting period. Twenty-one days after the start of the procedure, the hens returned to *ad libitum* feeding. Throughout the whole procedure, the animals had unlimited access to water and they were supplemented with 40 g of lime (solid calcium) for the period of food deprivation to support their bones, and prevent leg fractures due to flow of calcium to support egg production. The fasting period resulted in complete cessation of egg production, shedding of feathers, and weight loss, which all recovered fully.

### Experimental setup

We started with 64 individually labeled laying hens, each accommodated in an individual cage. At 21 months of age, the hens were randomly assigned into a control, *ad libitum*, group, and molted group, which resumed *ad libitum* feeding after the procedure. Laying efficiency and body weight were similar between the groups before the procedure (Supplementary Table 2). Molting resulted in complete sterility and about 30% loss of body weight. After the molt, the transient for full recovery took another couple of weeks, so a steady state was achieved only at 24 months of age. All 32 animals that went through the molt remained alive and healthy throughout the procedure, at least until steady state at 23 months of age. However, three hens remained completely infertile for an unknown reason, but other than that looked healthy and did not show any signs of stress or unusual behavior. While the qualitative conclusions of this study are valid with these infertile animals, this study focuses on the biology of responding hens. Therefore, the three non-responders were excluded from the analysis. All other 29 hens resumed laying potency of at least 40% and were all included in the study.

Laying efficiency of a hen (sample) is calculated per month, as the total number of laid eggs divided by the number of days. Median laying efficiency of a cohort is calculated monthly by first finding the median production of the cohort, then dividing by the number of days. When we correlated true-to-predicted laying efficiency, the true laying efficiency was calculated implicitly for the month that the hens were sampled.

Figure 2A follows the effect of molting on the performance of the whole population of the two groups. To do this, dead hens must also be considered, as ignoring individuals that left the cohort (i.e., dead) would present a bias towards strong animals rather than representing the whole diversified population.

We defined an animal as sterile after three consecutive weeks of ceasing egg production. In our facilities, there is no recovery under standard conditions after such a stretch of no production. In our hands, some animals can undergo periods of no production and recover even after a week of sterility, especially if the low efficiency is a consequence of heat stress during the summer. In the vast majority of case, 3–4 weeks of sterility are followed by mortality as described in the “Results” section.

### Blood sampling

From each layer, 1 mL of blood samples was taken from the wing vein and immediately added to a solution of 100 μL heparin-PBS (Sigma) at 10 mg/mL to prevent clotting. Samples were kept on ice for about 1 h and then centrifuged for 20 min at 10,000 rpm and 4°C in a refrigerated tabletop centrifuge. Plasma samples were aspirated to new tubes (Eppendorf 2 mL, safe lock 0030120094), froze immediately in liquid nitrogen, and stored at −80°C.

### Python programming and code deposit

Unless specified explicitly, the statistical analysis and computational work were done using Python programming language (version 3.9). All machine learning analyses were done using scikit-learn package, version 1.0.1. Mann–Whitney *U* test and permutations statistics were done using scipy version 1.7.2 and numpy 1.21.4. FDR correction was calculated using statsmodels package, version 0.13.0.

### Selection of hens for metabolomics

Note that metabolomics profiling was performed for all samples together after concluding the experiments, so individual performance and mortality were already known. Due to financial constraints, we selected 12 hens from each group and time point for metabolomics.

We set the baseline for evaluating metabolic age according to the control group. To do this, we randomly selected 12 hens at four time points: 21 months just before molt, 23 months, 27 months, and 33 months. A random selection of controls reflects the metabolic aging of untreated population.

As for the treated hens, to validate the efficiency of the treatment, we selected a subset of 12 hens that were less healthy than controls prior to molting, at 21 month of age, namely at t21 (Fig. 2C). Specifically, we excluded the top 10% of egg-producing animals, and randomly selected 12 animals from the remaining lower 90% of the population. The top 10% of producing animals consisted of animals with about 100% laying efficiency, and we did not want to test the effect of molting on these extremely healthy hens. This selection process resulted in lower initial efficiency of treated hens (Fig. 2C). We then followed the same hens longitudinally to directly associate any improvement in health to the treatment rather than population variability. By excluding the top 10% of animals based on their production prior to molting and randomly selecting only 12 hens from the remaining group, the heterogeneity of the initial pool of these treated animals is reduced compared to the control group. This reduced heterogeneity is demonstrated by a lower level of initial metabolic or reproductive noise, as shown in Fig. 6.

In this scheme, better performance after molt could not be attributed to better initial health, and the longitudinal tracking of the same individuals implies that any beneficial effect would be a consequence of molting procedure rather than population variance.

### Cytokine array

Chicken sera and their cytokine profiles were analyzed with a semiquantitative chicken cytokine antibody array that detects 10 chicken cytokines in one sample as in [8] (RayBio G-Series Chicken Cytokine Array 1, Raybiotech, Norcross GA USA https://www.raybiotech.com/chicken-cytokine-array-gs1-en, October 2021).

### Statistics

#### Aging biomarkers

The aging biomarker identification strategy is incorporated in the main text.

In addition to using Mann-Whitney with FDR correction using a cutoff of 0.1, we validated the statistical procedure by two independent experimental methods: first, a validation control cohort at t23 (Supplementary Fig1C, D). These control animals were deliberately excluded from the statistical analysis aimed at identifying biomarkers, resulting in the formation of a sound test cohort. Second, the biomarkers predicted the correct temporal behavior of molted animals, which could be used as a completely independent validation group for this matter (Fig. 3C, D).

#### Laying biomarkers

The laying biomarker identification strategy is incorporated in the main text.

The use of PCA or machine learning algorithm to test the collective strength of the markers results from the fact that individually, due to variance between animals of the same age and the complexity of aging, no individual marker could separate well between individuals. However, in an analogy to the “wisdom of the crowd” principle, since the markers are independent, their collective predictions were much better than the prediction of any individual biomarker.

#### Significance tests

Student *t*-test associated with laying efficiency was applied to the total number of eggs laid per hen in a month (0–31 per animal). We subsequently compared the two groups based on the number of eggs laid by each individual. Since each group has about 30 hens, we have egg production of 30 independent individuals per group for the *t*-test.

Random permutation was always carried out by permuting the animals and counting the number of events by which the difference between the median of control and treated animals in the permuted configuration is equal to or bigger than the difference in the original groups. This number was then normalized by the total number of permutations to get the *p*-value. Significance for Fig. 6B inset was calculated by permuting the animals and testing the number of events by which the difference between the permuted control-to-treated slope is equal or higher than the original, unpermuted, differences in the slope.

#### Data normalization

Due to orders of magnitude differences in the expression values among the various metabolites, we used data normalization for principal component analysis (PCA). Normalization was done on each metabolite to adjust its mean to zero and standard deviation to 1.

#### Noise statistics

The noise, CV, for egg production per group was calculated as the standard deviation, σ, over the mean, μ, for each month. As this was a longitudinal study, daily egg production for each individual was obtained throughout the study. Thus, at each month, the CV for each group was calculated where σ and μ were based on individual monthly laying efficiency.

To obtain significance for noise in egg production, we first noticed that the CV in molted hens resumes its initial value only 7 months after the procedure. We compared the slope in this 7-month period between control and molted cohorts. Since egg production is followed longitudinally over the same animals, we used paired-based permutation and permuted the values only within the same hen. We then recalculated the slope for the CVs within these 7 months, and compared the difference between the slope, the permuted control, and molted hens to the difference in the reference values before molt.

The noise for a given metabolite in a group was calculated as in [6]: the standard deviation, σ, divided by the mean, μ. This value is also referred to as the coefficient of variation, CV, for this metabolite. Thus, for each age group, we had 693 CVs, corresponding to the 693 metabolites. The representative noise of a group was considered the median of the CVs in the group. To obtain significance for the difference between 2 noise histograms, we used random permutation statistics, by permuting the labels (hens) of each group. To obtain significance for the behavior of the medians, we used random permutation of the labels and counted the number of times (of 1000 permutations) that the permutated set had the same or higher difference between of the medians compared to the original groups.

#### Machine learning implementation

Predicting metabolic age and laying efficiency was carried out under random forest regressor (RFR). To validate control hens at time point t23 or define the metabolic age of molted animals, the model was trained exclusively on the biomarkers obtained from the control data at time points t21, t27, and t33. Then the model was validated over the desired data set for predictions. To predict laying efficiency of molt hens, the model was trained on control and predictions were ran for molt data. Predictions for laying efficiency of control under laying and aging biomarkers were performed using leave-one-out scheme under RFR. Additionally, to improve performance, we stacked LASSO and RFR together.

#### Identification of common markers for aging and reproduction

Common markers for aging and reproduction were features that appear both  in the list of 212 aging biomarkers and in the list of 189 reproduction biomarkers, and were also consistent with molting slowing down aging and improving performance.

Of the features that were found, α-ketoglutarate (AKG) stood out, as it has been linked to extending lifespan in model animals [9–12]. To further confirm the role of AKG in molting, we run pathway enrichment analysis on the 212 aging and 189 laying biomarkers (MetaboAnalyst platform).

#### Metabolite extraction

Extraction and analysis of lipids and polar/semipolar metabolites were performed as previously described in the works of Malitsky et al. (34) and Zheng et al. (35) with some modifications that are described in detail in [6].

#### Liquid chromatography–mass spectrometry for lipidomics analysis

Lipid extracts were analyzed using a Waters ACQUITY I Class UPLC System coupled to a mass spectrometer (Thermo Exactive Plus Orbitrap) which was operated in switching positive and negative ionization mode. The analysis was performed using ACQUITY UPLC System combined with chromatographic conditions as described in [6].

#### Lipid identification and quantification

Orbitrap data were analyzed using LipidSearch software (version 4.2; Thermo Fisher Scientific, Waltham, MA). The validation of the putative identification of lipids was performed by comparing to the home-made library which contains lipids produced by various organisms and on the correlation between retention time (RT) and carbon chain length and degree of unsaturation. Relative levels of lipids were normalized to the internal standards and the amount of plasma used for analysis.

#### Liquid chromatography–mass spectrometry polar metabolite analysis

Metabolic profiling of the polar phase was done as described by Zheng et al. (35) and more specifically in [6].

The data processing was done using TraceFinder version 4.1 SP3 (Thermo Fisher Scientific) when detected compounds were identified by accurate mass, RT, isotope pattern, fragments, and verified using an in-house-generated mass spectra library.

## Results

### A transient pause of reproduction promotes reproductive plasticity and health and reduces mortality in old hens

**Fig. 1:**
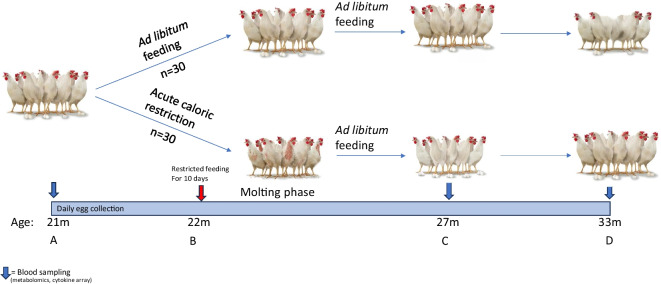
Illustration of an aging-driven induced molting cycle in hens. **A** At 21 months of age, reproductive aging lowers egg production, such that the flock becomes commercially unsustainable. **B** At 22 months of age, animals were randomly assigned into control, *ad libitum* fed hens, and treated hens, which underwent acute and sever caloric restriction protocol. The protocol resulted in shedding of feathers (i.e., molting) and complete cessation of reproduction. Note that in reality, the shedding of feathers does not result in nakedness, and the patchy bald areas are for illustrative purposes. **C** At 27 months of age, control hens continue to age and display a progressive decline in egg production. However, following the molt, and upon resuming *ad libitum* feeding, treated hens undergo a renewal of feather growth and reproductive plasticity as laying efficiency recovers almost to peak production. **D** At 33 months of age, both groups display progressive aging phenotype. However, post-molt recovery results in lower mortality and higher reproduction of treated animals

A schematic illustration of an aging-driven induced molting cycle and its comparison to control is shown in Fig. 1. At 21 months of age, the flock demonstrates variable aging phenotype [13] with an overall progressive and systemic decline in animal health, number of eggs laid, and egg shell quality (A). A transient caloric restriction protocol is applied, resulting in shedding of feathers (i.e., molt) and sterility (B). Once the molt is complete, the flock gradually resumes *ad libitum* feeding, resulting in rapid regrowth of feathers and reproductive plasticity, where egg production (and egg shell quality) recovers almost to its maximal rate. Control animals continue to age and display a progressive decline in reproduction (C). At 33 months of age, both groups display notable aging phenotypes. Nevertheless, treated hens appear younger and exhibit higher reproduction relative to control (D).

We started by validating the main characteristics of molting, focusing on egg production, mortality, and body weight [3–5].

**Fig. 2:**
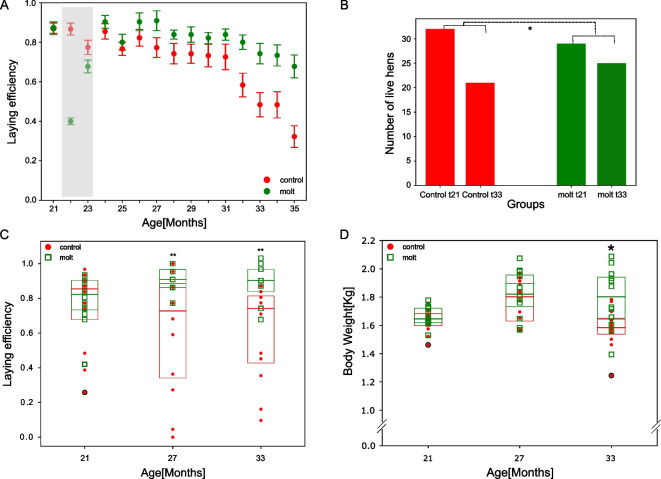
**A** Molting improves reproduction. Median laying efficiency of molted (green) and control (red) groups. Error bars are SEM for laying efficiency. Molting started at 22 months of age and continued through 23 months of age. The gray rectangle labels the transient period until reproduction, feathering, and body weight recovered at 24 months of age. BW of control (1688 ± 156)g, and molt (1763 ± 177)g for (average ± SD) before the procedure were not significantly different. Median efficiency at each age is calculated for the entire 32 (29) animals that composed the original control (molted) group. **B** Molting reduces mortality. Mortality of control (red) and molted (green) groups throughout the study. Control hens lost 34% of the population, while molted group 13%.* denotes *p* < 0.05; *p* = 0.013 for the difference in survival values, significance was calculated using two-sided binomial test. **C** Molting improves reproduction of the metabolomics-selected hens. Laying efficiency of the molt (green) and control (red) groups at 21 months (before molt), 27, and 33 months of age presented as boxplots. Red dots and open green squares represent individuals of control and molted cohorts respectively. Each cohort consisted the 12 animals that were also sampled for serum metabolomics. ** denotes *p* < 0.01 (under *t*-test, calculated according to the total raw number of monthly egg production across individuals—see the “Methods” section). **D** Molted hens have a higher body weight than controls in old age. Body weight of control (red) and molted (green) groups at 21 months (before molt), 27, and 33 months of age depicted as boxplots. Red dots and open green squares represent individuals of control and molted cohorts respectively. * denotes *p* < 0.05 (under *t*-test)

Figure 2 illustrates the effect of molting on egg production (2A) and mortality (2B) for the control and treated hens (see methods). Figure 2C and D illustrate the effect of molting on two subsets consisting of 12 individuals each, derived from a control group and a treated group, which were chosen for metabolomics analysis (see the “Methods” section).

Consistent with previous reports [14], molted hens show increased reproduction, lower mortality, and even a higher body weight in old age than control (Fig. 2D).

Increased efficiency and body weight after the molt, when the animals resumed AL feeding, suggest a more efficient and possibly younger metabolism induced by the procedure. Last, we tested whether improved health can result exclusively from the cessation of reproduction. In the untreated control cohort, 14 of the 32 animals became sterile during the study. Of these, 10 cases were followed by mortality (out of 11 deaths), whereas only four of the surviving 21 hens were sterile (*p* = 0.0001, Fisher test). Therefore, naturally pausing egg production is an indication of frailty rather than improved health.

### Molting slows down metabolic aging

Next, we assayed for systemic molecular effects of molting on aging. Since metabolism becomes inefficient with age [15], we assessed the effect of molting on metabolic aging, using metabolomics profiling from the circulating plasma of live hens [6].

We, therefore, set out to find molecular aging biomarkers in order to model the metabolic age of individuals. Animals were sampled at 3 time-points: t21, at 21 months of age, just before molt; t27, at 27 months of age; and t33, at 33 months of age. Control hens were also sampled at 23 months of age, t23, to be used as a validation cohort for aging biomarkers. Consequently, these control animals were excluded from the statistical analysis aimed at identifying biomarkers to generate a reliable test cohort. To generate aging biomarkers, we focused exclusively on control hens, as these set the baseline for aging in the reference population. We, therefore, randomly selected a subset of 12 animals at each time point from the control cohort as a representative sample. On the other hand, to ensure that the beneficial effects of molting are not attributed to improved baseline health relative to controls, we excluded the top 10% of egg-producing animals (100% efficiency) of the molting cohort at 21 months of age, prior to the initiation of molting, and randomly selected a subset of 12 animals from the remaining population. Indeed, these animals exhibited lower performance with respect to controls (Fig. 2C and the “Methods” section). We further tracked the same animals longitudinally, so that any changes in metabolism could be directly linked to molting and would not be affected by population variability (see Methods).

The aging biomarkers were determined based on three time points, i.e., t21, t27, and t33, from the list of 693 metabolic features as follows: First, for a given pair of time points (e.g., t21–t27), a two-tailed Mann-Whitney test was utilized to obtain a *p*-value for each individual feature. Subsequently, the list of *p*-values was FDR corrected (Benjamini–Hochberg, [6]) using a cutoff of 0.1. This resulted in a list of significant features for the given pair of time points. We then used an OR condition to generate a pool of possible markers; namely, we pooled all the significant features that appeared even in one of the three possible pairs, t21–t27, t21–t33, and t27–t33. Lastly, we removed from the list any features whose median values were not monotonic with time. Thus, the final list of 212 aging biomarkers included features that change monotonically with age between the three time points and exhibit significance in at least one pair of time points (Supplementary Table 1).

**Fig. 3:**
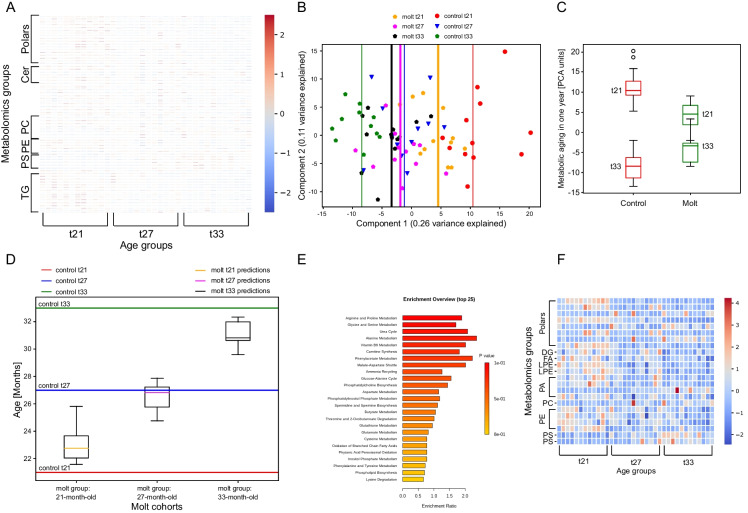
**A** Temporal behavior of the aging biomarkers illustrated as a heatmap. The three age groups, t21, t27, and, t33, of control hens are clustered in the *x*-axis, and the metabolites in the *y*-axis. Each row represents a metabolic feature and each column represents an individual hen. Only chemical groups that have more than 10 metabolites are shown explicitly. The markers are spread all over the metabolome (Supplementary Table 1). Number of animals for each group, *n* = 12. Blue (red) indicates low (high) relative expression. Expression levels for each feature were normalized to have mean = 0 and a standard deviation of 1. **B** Metabolic aging biomarkers project the correct temporal hierarchy and a slower aging rate for molted hens. Principal component analysis (PCA) of individual samples according to age groups based on 212 aging biomarkers, for control and molted animals. Shown are the two PCs with the largest variance (29% and 11% for PC1 and PC2, respectively). Vertical lines represent the medians of the corresponding groups. Red, blue, and green dots (and vertical lines) correspond to the 36 samples of control groups. Gold, purple, and black dots (and vertical lines) correspond to the 36 samples of the molted groups. The projections maintain the correct hierarchy of the molted animals. In the case of molted animals, the projections correspond to longitudinal tracking following the same animals in the three time points. On the main axis, which represents metabolic aging, the molted group appears to have an initial older metabolic age and a younger final age relative to control, indicating slower metabolic aging. **C** Metabolic aging of control (red) and molted (green) animals over one chronological year according to the first PCA component (panel **B**), illustrated by boxplots. The representative metabolic aging for each group between t21 to t33 is represented by the difference between he medians of each cohort (*p* = 0.0094, random permutations). **D** Random forest regressor predicts a slower metabolic aging rate for molted hens. Horizontal lines indicate the correct age of control hens at time-points t21 (red), t27 (blue), and t33 (green) that were the reference for selecting the aging biomarkers. The three box plots refer to the predicted age of 12 individually tracked hens of the molting cohort. The same molted hens were sampled at 21, 27, and 33 months of age, resulting in three samples per live hen. The initial median predicted age is 23 months (solid gold line within the box), and the last median age is 31 months (solid black line). The box plots show no outliers and illustrate the correct hierarchy within the molted cohort. The model was trained exclusively on control hens, and predictions were given for all 36 samples of the 12 molted animals. While control hens aged by 12 months, molted hens aged by 8 months according to the biomarkers. **E** Top hits of metabolic pathways based on aging biomarkers. The list of the metabolic biomarkers was run through MetaboAnalyst platform for enrichment analysis. **F** A heatmap of common biomarkers of aging and reproduction (Supplementary Table 1), presented as in panel **A**. The *y*-axis represents the chemical groups of the markers

Figure 3A presents the temporal expression pattern of the markers as a heatmap. The heatmap shows that the aging biomarkers are spread all over the metabolic chemical groups. Further, while most markers clearly distinguished between two age groups, none appeared as a clear-cut separator between all three time points. Therefore, we wanted to test whether, collectively, the 212 aging biomarkers can separate well between all three age groups.

Supplementary Figure 1 summarizes the collective power of the biomarkers. First, PCA projections and random forest classifier validated that the markers can assign the correct age group to control hens at t21, t27, and t33 (Supplementary Figure 1A, B). Second, PCA projection and random forest regressor assigned the correct age to the validation cohort, at time t23. The regressor assigned a median age of 24 months, where all hens were within the normal range as seen in the corresponding box plot (Supplementary Figure 1C, D). Thus, the metabolic aging biomarkers evaluated well the true age of the control cohort.

We then tested the predictions of the aging biomarkers obtained exclusively from control groups on molted animals. Figure 3B presents PCA projections at t21, t27, and t33 of control and molted animals. The projection assigns the correct time hierarchy for molted hens, indicating that the markers also represent aging in molted hens. In addition, the distance on the main axis between t21 to t33 in molted animals is smaller than the distance in controls, indicating slower metabolic aging. Lastly, the initial selection of hens with relatively lower health status in the treated group is represented by the first principal component (PCA1) in Fig. 3B: the initial metabolic age of molted hens is older than the controls (Fig. 3B, age is reflected in the PCA1 axis in right-to-left directionality). However, molting slows down metabolic aging, so treated hens are metabolically younger than controls after one chronological year (Fig. 3B, C).

We subsequently assessed explicitly the difference in metabolic aging based on the main PCA axis as a proxy for metabolic age (Fig. 3C). By utilizing the medians of each group as representatives of metabolic age (as depicted in Fig. 3B), molted hens underwent a significantly slower metabolic aging relative to control over one chronological year, namely between t21 and t33 (Fig. 3C, *p* = 0.0094, random permutation).

Figure 3D illustrates the quantitative age assigned by random forest regressor at t21, t27, and t33 to molted groups after training exclusively on control animals. The quantitative model exhibits the correct age hierarchy in molted groups, predicts a slower aging rate of about 25%, and assigns an initial older age to molted animals with respect to controls. The box plots also indicated that all molted animals were within the normal range without any outliers. To establish a connection between aging biomarkers and overall metabolism in vertebrates, we sought to identify the metabolic pathways in vertebrates that are enriched by the biomarkers. Figure 3E illustrates the top hits of pathway enrichment analysis based on the aging biomarkers using the MetaboAnalyst platform. These findings reveal that all of the top enriched pathways are fundamental in vertebrates, for example, amino acid metabolism and the urea cycle. In laying hens, daily egg production establishes a robust connection between aging and reproduction. Consequently, we aimed to identify specific metabolic features that are conserved among vertebrates and hold significance in aging and reproduction. Figure 3F presents a collection of 23 features that appear both in the list of aging and reproductive biomarkers in the form of a heatmap (see the next section and the “Discussion” section).

In summary, the aging marker set shows a slower rate of metabolic aging for molted hens.

### Laying biomarkers indicate a younger reproductive age for molted animals

Laying an egg daily allows a clear distinction in reproduction even between individuals of the same age. We, therefore, wanted to develop metabolic laying biomarkers based on circulating plasma to quantify the reproduction of individuals.

Biomarkers of reproduction were derived using Spearman correlation and FDR correction for each metabolite across the 47 control hens. Specifically, we did the following: (i) For each metabolic feature, we extracted its values from the profiling dataset (metabolomics Tables) for each of the 47 control hens. (ii) We calculated the monthly reproductive efficiency of each of the 47 hens based on the month in which they were blood sampled. This allowed us to obtain both the metabolic expression profile and corresponding reproductive efficiency of each hen explicitly at the time of blood sampling. (iii) We applied Spearman correlation, along with its *p*-value, between the expression values and reproductive efficiency across the 47 hens for each metabolite. After correcting for multiple hypotheses using FDR, a list of 189 significant biomarkers was obtained.

**Fig. 4:**
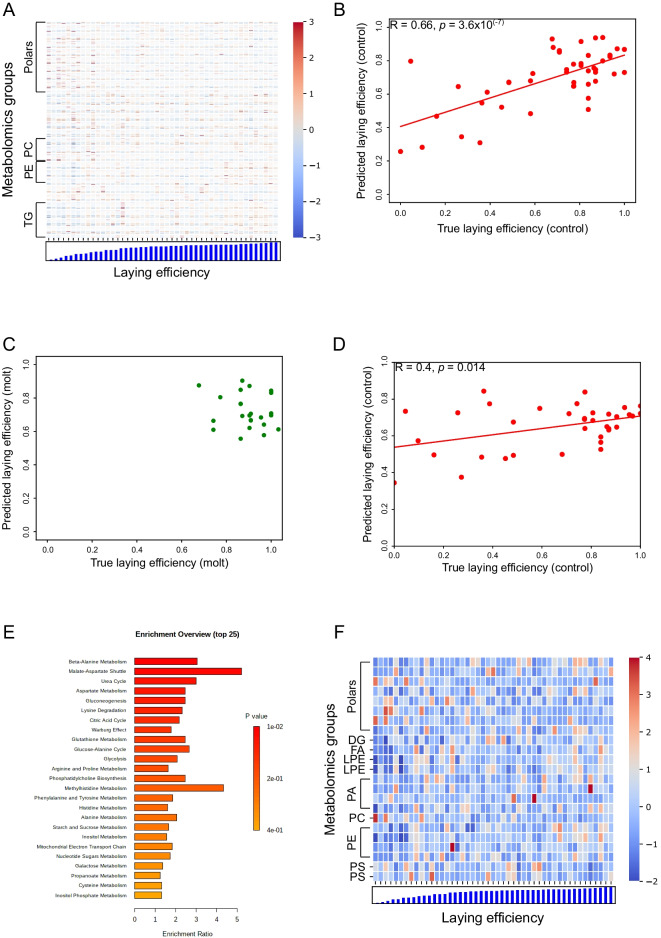
**A** Correlations between individual laying biomarkers and laying efficiency illustrated as a heatmap. Each row represents the expression of the corresponding metabolite. Bars at the bottom illustrate are monthly laying efficiency of individual animals at the age of blood sampling. Therefore, each column represents expression values of all laying biomarkers of the same individual, whose efficiency is presented at the bottom. Implicit metabolite groups are shown only for chemical groups that have more than 10 features. Note that the markers are spread all over the metabolome (Supplementary Table 1). Number of animals, *n* = 47. Color bar on the right side: Blue (red) indicate low (high) relative expression. Expression levels for each feature were normalized to have mean = 0 and standard deviation of 1. **B** Metabolic laying biomarkers predict the reproductive efficiency of control hens. Predicted laying efficiency (*y*-axis) vs. true laying efficiency of 47 control hens at time points 21, 23, 27, and 33 months. Predictions are based on 189 laying biomarkers and calculated using leave-one-out scheme. Each point represents an average efficiency of individual hens over the corresponding month. The prediction was calculated using leave-one-out scheme. The red line represents a linear regression, *R* = 0.66 (Pearson), *p* = 3.6×10^(−7)^. **C** Metabolic laying biomarkers predict the correct range of laying efficiency in molted hens. Predicted laying efficiency (*y*-axis) vs. true laying efficiency of 24 molted hens at time points 27 and 33 months, i.e., post molting. Predictions are based on 189 laying biomarkers identified from the control group. The model was trained exclusively on control hens and used for predictions on molted animals. Each point represents an average efficiency of individual hens over the corresponding month. **D** Metabolic aging biomarkers predict the laying efficiency of control hens. Predicted laying efficiency (*y*-axis) vs. true laying efficiency of 36 control hens at time points 21, 27, and 33 months. The predictions were based on the 212 aging biomarkers using leave-one-out scheme. Each point represents an average efficiency of individual hens over the corresponding month. The prediction was calculated under RFR using leave-one-out scheme. *R* = 0.4 (Pearson), *p* = 0.014. **E** Top hits of metabolic pathways based on the biomarkers of reproduction. The list of the metabolic biomarkers was run through MetaboAnalyst platform for enrichment analysis. **F** A heatmap of common biomarkers of aging and reproduction (Supplementary Table 1), presented as in panel **A**. The *y*-axis represents the chemical groups of the markers

Figure 4A presents the correlation between individual reproductive markers and reproductive efficiency as a heatmap. Similar to the aging biomarkers, biomarkers of reproduction are spread all over the metabolome, and no individual marker appears to predict well the efficiency of the 47 animals.

We, therefore, applied linear regression to test the collective predictive power of all markers. Figure 4B shows a significant correlation between the true value and the predicted value of reproductive efficiency (*R* = 0.66, *p* = 3.6×10^(−7)^). The predictive power of the blood-based markers is independent of age, where either young or old hens fit the model to the same extent (Supplementary Figure 2A). Additionally, the fertility markers can distinguish between individuals of the same age (Supplementary Figure 2A). In terms of identifying animals with above 50% efficiency, the model captures 97% of this group, proving a practical tool for breeding selection (Fig. 4B).

We then set out to test the ability of the laying biomarkers to predict the efficiency of molted hens. However, the variance in the molting group between t27 and t33 is very low (both groups have a median of 90% efficiency), so we needed the markers to identify the correct range of production. Indeed, Fig. 4C shows that the laying biomarkers selected and trained exclusively on control hens predicted the correct range of reproduction for molted animals. Last, as aging and reproductive aging are linked, we tested the predictive power of the aging biomarkers in predicting laying efficiency and found a significant correlation (*R* = 0.4, *p* = 0.014) between actual and predicted reproduction (Fig. 4D). This prediction is not biased by age group (Supplementary Figure 2B). Figure 4E illustrates the top hits of pathway enrichment analysis based on the reproduction biomarkers using the MetaboAnalyst platform. These findings reveal that all of the highly enriched pathways are fundamental in vertebrates, for example, the urea cycle. The 23 markers that are common to aging and reproduction are shown in Fig. 4F as a heatmap (see also the “Discussion” section).

### Molting slows down aging in the immune system

**Fig. 5:**
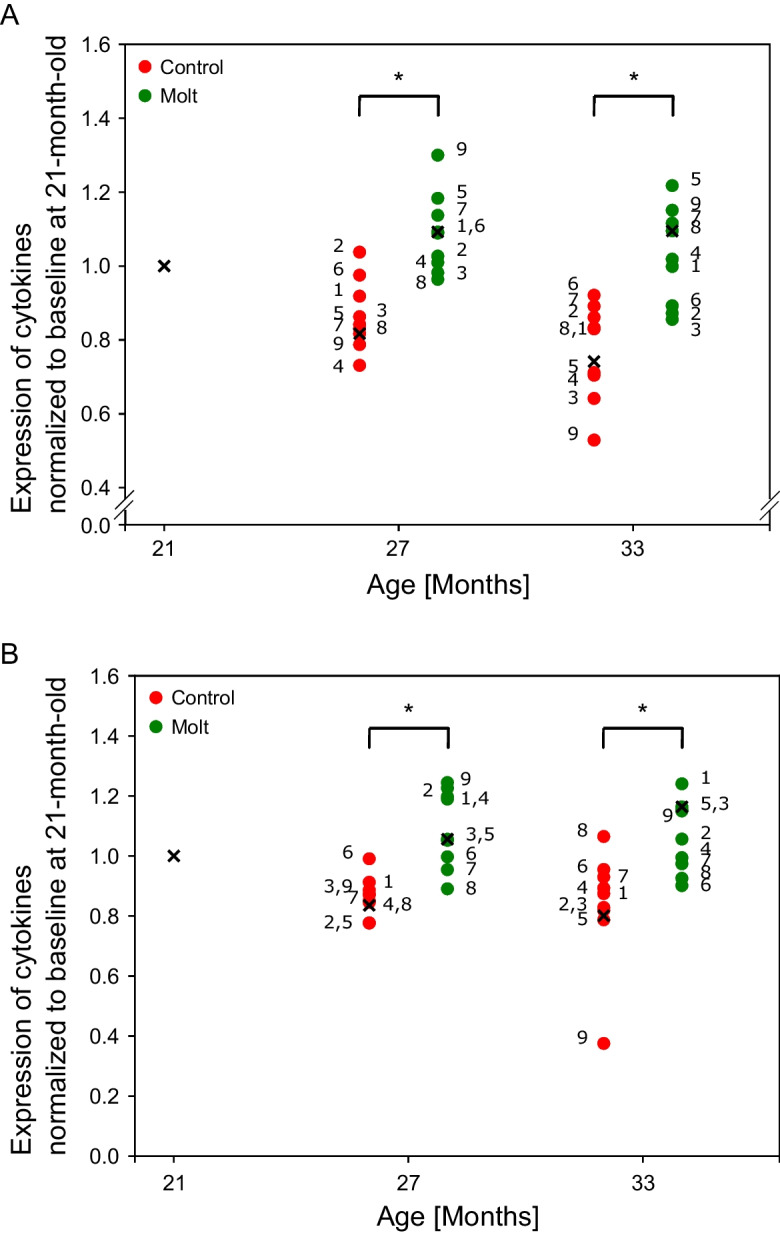
**A**, **B** Cytokine expression indicates that molting slows down immunological aging. Cytokine levels for control (red) and molted hens (green) as a function of age. Each dot represents an element of the array: the annotations of individual elements [1–9] correspond to [IFNg, IL-6, IL-10, IL-12p40, IL-16, IL-21, Netrin, Pentraxin, Rantes]. The data correspond to time points t21, t27, and t33. The separation of the control and molted cohorts around time points t27 and t33 is employed to improve visual clarity. Each cytokine is an average over four animals, and data is normalized by the initial values at t21. The medians of all nine array elements for each age and group are depicted as black “*x*” symbols, indicating their normalized values relative to their baseline values at t21. At 27 and 33 months of age, levels of molted hens are higher than control, indicating a younger immunological age. Panels **A** and **B** correspond to two biological repeats of the experiment. * denotes *p* < 0.05 for control vs molted hens. Significance was calculated using a random permutation test (permuting the hens) and comparing the levels of the medians between control and molted groups of the same age

The immune system is strongly affected by aging [16]. We used an array-based assay (RayBiotech, GSG CTY-1, [8]) to measure the expression of nine immuno-proteins in the blood directly. Since seven of the nine proteins are cytokines, we will refer to the array as a cytokine array. Subsequently, the median expression of the nine array elements was employed as a representative measure of the cytokine array for every time point and cohort. Figure 5A and B summarize data from two repeats at t21, t27, and t33 for control and molted hens; first, cytokine expression decline with age. Second, there is an increase in immunological values due to molting, suggesting that molting rejuvenates the immune system (Fig. 5A, B). Last, at the same age, the molt cohort appears significantly younger than the control indicating younger immunological age (*p* = 0.04 under random permutation test). Taken together, the data obtained from the cytokine array suggest that molting slows down or even reverses immunological aging.

### Molting reduces heterogeneity between individuals

A common aspect of aging is increased heterogeneity between individuals [6, 17, 18].

This property has been demonstrated in many organisms and multiple tests, such as gene expression, metabolism, and cognition [6, 18–20]. In particular, an increase in heterogeneity with age was linked to an increase in frailness in humans [21].

We have previously introduced a simple and straightforward measure of heterogeneity of a feature in a population, noise, defined as its standard deviation normalized by the mean, namely the CV [22]. Indeed, noise has been shown to be a universal metabolic marker of aging in hens, humans, and mice [6]. Because aging is a complex phenomenon affected by many pathways [23], noise of an individual feature may not be sufficient to capture aging rates between various treatments. Therefore, we set out to use noise of the complete metabolome [6], the median of the set of CVs of all features, as a robust marker evaluating aging rates of molted and control groups.

**Fig. 6:**
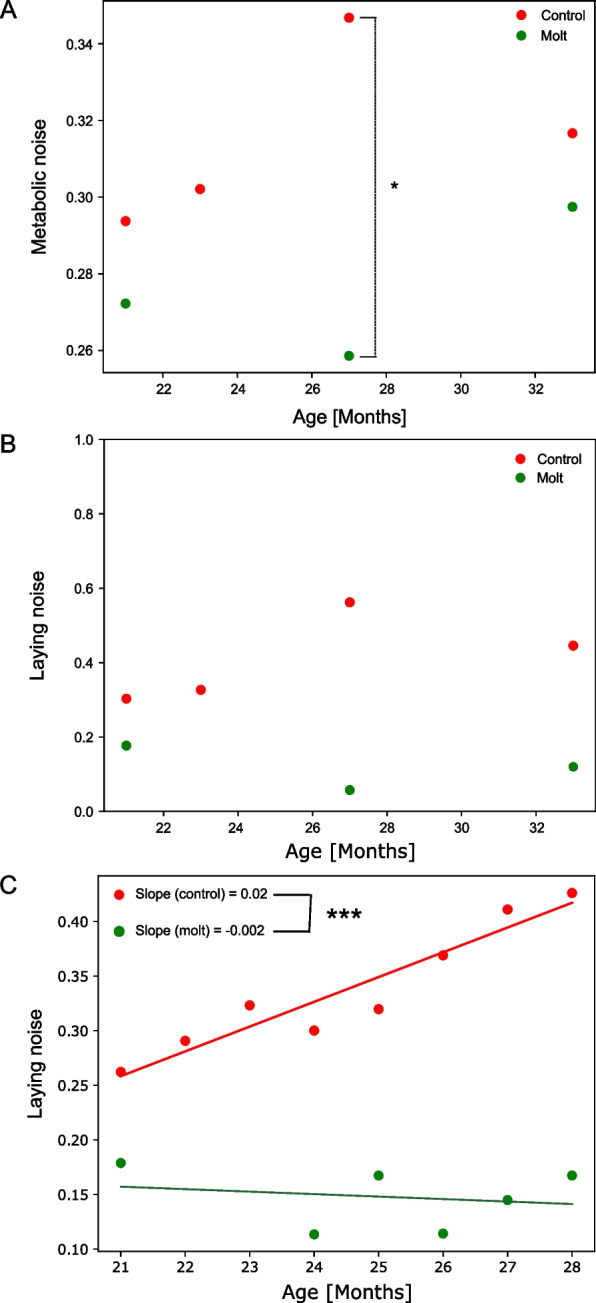
**A** Noise vs. age of the metabolome (**A**) and egg production (**B**) for the 12 animals that were blood-sampled for metabolomics. Control animals (red) are also in a steady state at 23 months, which is still a transient period for molted animals (green). Metabolic noise is defined as the median of the set of CVs obtained from all metabolites for a specific group and specific age. * denotes *p* < 0.05. Significance was calculated using a random permutation test (permuting the hens). **C** Reproduction noise for the entire cohort. In the first 7 months after the molt control hens exhibit a monotonic increase with noise. However, molted hens show a slight negative slope. In this period, all hens in the study are still alive. Red and green lines indicate linear regression of the corresponding groups. *** denotes *p* < 0.001 for the difference between the slopes. Significance was calculated using a random paired permutation test (permuting values exclusively within each hen)

Figure 6 illustrates how noise changes due to molting: First, molting reduces the heterogeneity of the metabolome (Fig. 6A, *p* = 0.01, random permutation test), as reflected by the decline in metabolic noise between t21, before the procedure, to t27. Control animals, however, exhibit a steady increase in metabolic noise during this period (Fig. 6A). Second, molted hens are always less heterogeneous than control. A reduction in noise at the last time point in the control group is similar to what is seen in demographic aging in older adults [24]; the higher mortality in the control group generates a selection for strong animals at old age, which increases homogeneity. Lastly, prior to molting, at 21 month of age, the treated group displays a reduced level of metabolic noise relative to the control group. This difference is attributed to a selection bias in the treated group for animals with lower performance (see the “Methods” section).

As aging rejuvenates the reproductive tract, we expected the procedure to reduce the heterogeneity of egg production in molted animals. Indeed, noise in egg production illustrated the same trend as in metabolomics (Fig. 6B). We also calculated the noise for the entire cohort as a function of age before mortality started to affect reproduction (Fig. 6C). Indeed, molting resets the reproductive tract, and the molt cohort becomes less heterogeneous than at its baseline value after the procedure for about 7 months (Fig. 6C, *p* = 0.0007, random permutation).

Taken together, reduced noise following the molt is indicative of reducing population variability, suggesting induced rejuvenation by the procedure.

## Discussion

This study presents the laying hen as a special model for reproduction and aging due to its exceptional laying efficiency. Utilizing this efficiency, we identified circulating biomarkers capable of accurately quantifying reproduction of individuals. In particular, we found that population variability leads to a low predictive accuracy of individual biomarkers. Thus, in order to attain accurate prediction of individual hen’s performance, it was essential to build a machine-learning-based model that integrates all available markers.

A notable success of the reproductive markers was capturing the correct range of efficiency in control and treated animals: efficiency of control hens ranged from zero to one, whereas efficiency following the molt ranged from 0.6 to one (Fig. 4).

One unique aspect of hens is their ability to rejuvenate their reproductive tract following a severe and acute caloric restriction protocol; a standard scheme used in agriculture to extend the profitable lifespan of the flock.

Here we used a variety of molecular, computational, and physiological assays to show that this procedure systemically slows down, even reverses, aging.

The process by which the biomarkers of metabolic aging and reproduction were initially selected and verified in a control cohort, and subsequently proved to be valid also on treated hens, is critical to identifying markers that are robust and reliable for identifying aging-related manipulations. Markers that are only tested on control, unperturbed animals, without validation against any intervention may be too specific and not suitable for generally distinguishing biological and chronological age.

Using control animals as a reference for identifying the biomarkers also facilitated the identification of a subset of metabolic changes resulting from molting that are specifically associated with aging.

In general, using a control group for determining the biomarkers and then comparing the behavior between controls and treated animals could be statistically flawed. Nevertheless, using FDR with a lower cutoff of 0.1 and two experimental validations, in particular the correct prediction of temporal hierarchy in molted hens (Fig. 3B, D), suggests that in our work the comparison is sound and the biomarkers are group independent.

Because reproductive plasticity is not common in vertebrates, and because commercial hens were intensively selected for extreme production, we were concerned that our biomarkers would not be relevant to aging in vertebrates. We, therefore, looked for features that appear both as aging and reproductive biomarkers, and were also consistent with molting slowing down aging and improving performance (Supplementary Table 1, Figs. 3F, 4F).

Of the common features, α-ketoglutarate (AKG, third marker from the top in Figs. 3F, 4F) stood out, as it has been linked to extending lifespan in model animals [9–12]. Pathway enrichment analysis (MetaboAnalyst platform) found that all top hits of aging and laying markers are associated with AKG (Figs. 3E, 4E). Finding a molecular marker at the nexus of aging and reproduction in hens, which is a key aging regulator in vertebrates further supports the laying hen as a model animal for aging and reproduction.

Of the other biomarkers, triglycerides and ceramides are strongly associated with longevity pathways [25]. For example, a deficiency in the triglyceride synthesis enzyme acyl-CoA:diacylglycerol acyltransferase 1 extended lifespan in mice, and inhibiting ceramides promoted longer lifespan in *C. elegans* and *D. melanogaster*. These data further support the relevance of hens as a valid vertebrate model. However, since the laying hen is not a genetic model animal, focusing on food supplements, such as AKG, appears as a practical approach to test the role of the promising candidates in promoting healthspan and reproduction.

Lastly, we presented metabolic noise, a unitless parameter that reflects the heterogeneity of the entire metabolome of a population, as a generic measure of aging. In control animals, metabolic noise increased with age, reflecting increased variability or entropy with time. However, molting reduced metabolic heterogeneity, suggesting a systemic rejuvenation. Metabolic noise, therefore, is a bona-fide marker of aging rather than reflecting temporal propagation of a system. Consistent with metabolic noise, molting reduced heterogeneity in reproduction, as expected by rejuvenating the reproductive tract. In this scheme, molting was used as a reference to test the robustness and reliability of metabolic noise as an aging biomarker that can reveal anti-aging treatments in vertebrates.

Because noise of individual features is unitless and reflects the relative deviation from the population mean, multiple features that span orders of magnitude can be combined to calculate system-wide noise, e.g., metabolic noise, as a single and robust number, to estimate the rate of aging.

Therefore, one can generate a set of noises for all quantitative features related to the agiome, all features relevant to aging. Then, the median of this set can represent the rate of aging of a population and can be used as a quick proxy for successful aging treatments, shortening the timeline for developing aging drugs.

### Supplementary information

ESM 1

ESM 2

ESM 3

ESM 4
