# Supplementary figures and images for: Reduction in metabolic noise reveals rejuvenation following transient severe caloric restriction
Source: GeroScience. 2023 Nov 10;46(2):2343–58. doi: 10.1007/s11357-023-00969-1 (PMC10828374; doi:10.1007/s11357-023-00969-1)

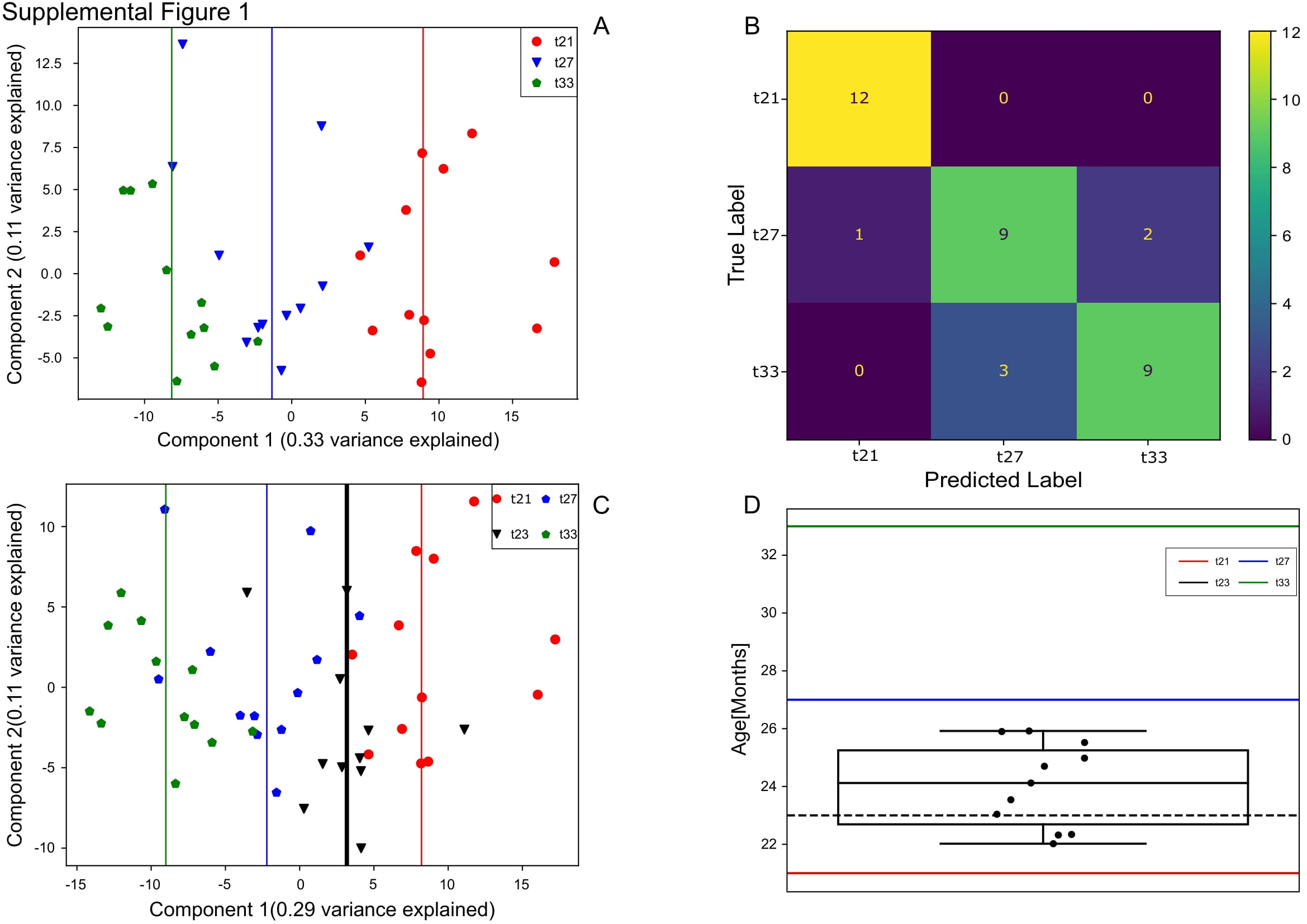

Supplement: Supplementary file 1 — ESM 1 [file 11357_2023_969_MOESM1_ESM.jpg]

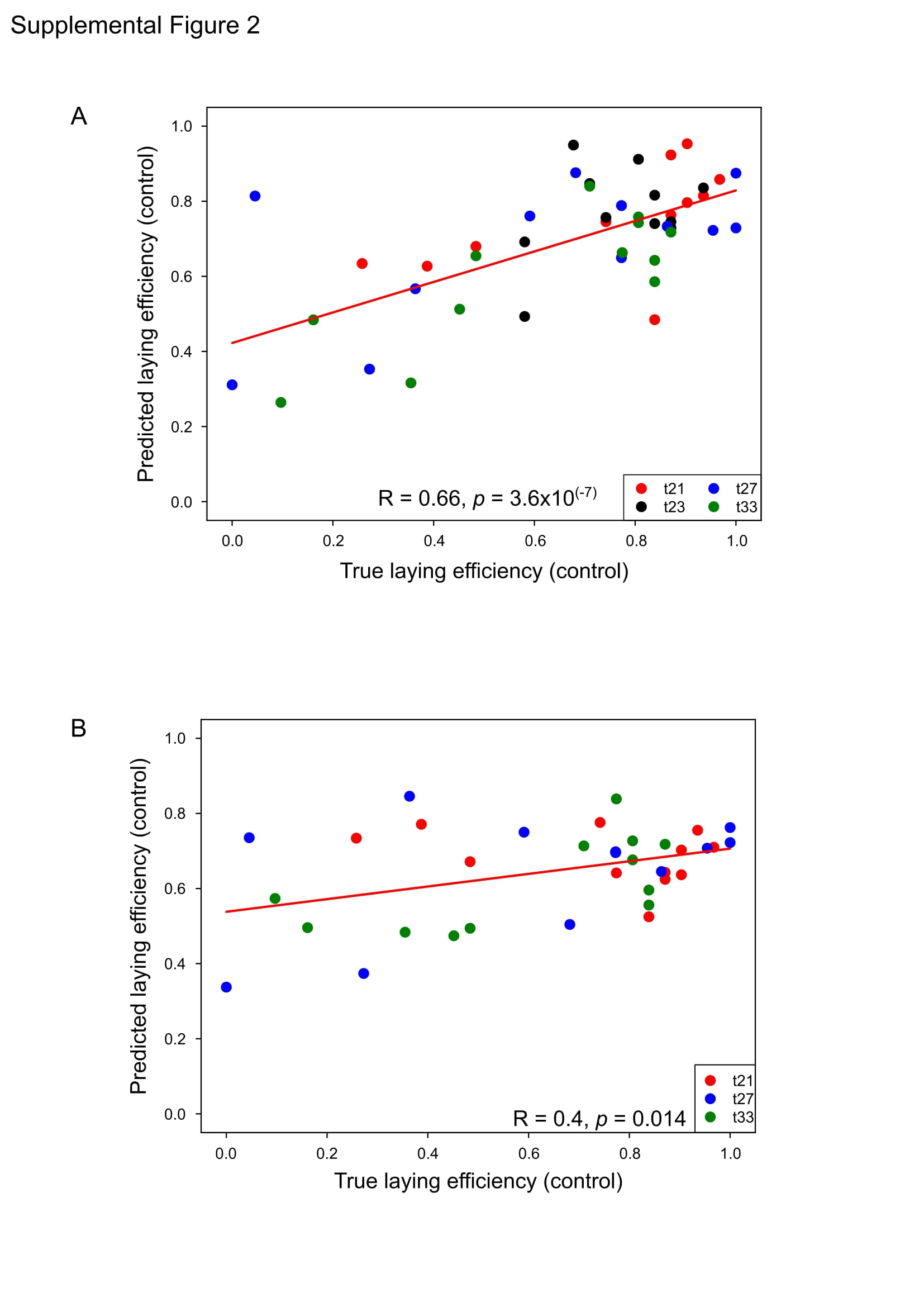

Supplement: Supplementary file 2 — ESM 2 [file 11357_2023_969_MOESM2_ESM.jpg]
